# Development of Three-Layered Composite Color Converters for White LEDs Based on the Epitaxial Structures of YAG:Ce, TbAG:Ce and LuAG:Ce Garnets

**DOI:** 10.3390/ma16051848

**Published:** 2023-02-23

**Authors:** Anton Markovskyi, Vitaliy Gorbenko, Tatiana Zorenko, Sandra Witkiewicz-Lukaszek, Oleg Sidletskiy, Alexander Fedorov, Yuriy Zorenko

**Affiliations:** 1 Department of Physics, Kazimierz Wielki University in Bydgoszcz, Powstańców Wielkopolskich Str., 2, 85-090 Bydgoszcz, Poland; 2 Mechantronic Department, Kazimierz Wielki University in Bydgoszcz, Kopernik Str., 1, 85-074 Bydgoszcz, Poland; 3 Institute for Scintillation Materials, NAS of Ukraine, 61001 Kharkiv, Ukraine; 4 Institute for Single Crystals, National Academy of Sciences of Ukraine, 61178 Kharkiv, Ukraine

**Keywords:** single crystalline films, liquid-phase epitaxy, mixed garnets, Ce^3+^ ions, composite film-crystal photoconverter, Planar-Chip-Level Conversion, white LEDs

## Abstract

This work was dedicated to the development of novel types of composite phosphor converters of white LED, based on the epitaxial structures containing Y_3_Al_5_O_12_:Ce (YAG:Ce) and Tb_3_Al_5_O_12_:Ce (TbAG:Ce) single crystalline films, steeply grown, using the liquid-phase epitaxy method, onto LuAG:Ce single crystal substrates. The influence of Ce^3+^ concentration in the LuAG:Ce substrate, as well as the thickness of the subsequent YAG:Ce and TbAG:Ce films, on the luminescence and photoconversion properties of the three-layered composite converters were investigated. Compared to its traditional YAG:Ce counterpart, the developed composite converter demonstrates broadened emission bands, due to the compensation of the cyan–green dip by the additional LuAG:Ce substrate luminescence, along with yellow–orange luminescence from the YAG:Ce and TbAG:Ce films. Such a combination of emission bands from various crystalline garnet compounds allows the production of a wide emission spectrum of WLEDs. In turn, the variation in the thickness and activator concentration in each part of the composite converter allows the production of almost any shade from green to orange emission on the chromaticity diagram.

## 1. Introduction

White light-emitting diodes (wLEDs) are now widely used in general lighting. Due to their advantages of high efficiency, low energy consumption, and low cost, wLED technologies have recently become a significant source of display backlighting, streetlights, headlights, and indoor and outdoor lighting [1]. Compared to conventionally used incandescent, fluorescent lamps, and high-pressure sodium vapor lamps, they offer faster switching times, energy savings, environmental protection, extended lifetimes, rich colors, and other benefits [2,3]. In phosphor-converted white LEDs (pc-wLEDs), a blue InGaN chip is directly packed with a phosphor converter, that is dispersed in silicon resin (phosphor-in-silicon; PiS) [4]. From its first commercial use in 1997 until today, YAG:Ce garnet is the most widely applied wLED phosphor [5]. The function of the phosphor converter is to absorb the blue light emitted by the LED chip and to convert it to yellow light, which can be achieved due to the Ce^3+^ ions’ luminescence. The combination of the transmitted blue light and yellow Ce^3+^ emission band, due to the 5d-4f transitions, allows the production of white light [6]. However, such a combination gives rise to rather cold white light, with the correlated color temperature (CCT) = 4000–7500 K, and a poor color rendering index (CRI), around 60, due to the red light deficiency. To improve the color temperature and make a warmer white LED, an orange or red emitting phosphor can be added [7].

With the increasing demand for high-brightness lighting, high-power wLEDs and white laser diodes (LDs) are rapidly developing. The operating temperature of a color converter can reach up to 200–400 °C under high-power LED or LD excitation. The heat generated from the LED chip and PiS (Stokes shift and to optical losses) cannot be efficiently dissipated because of the poor thermal stability and weak thermal conductivity of the resin (<0.5 Wm^−1^K^−1^) [8]. With the increase in the wLED temperature, color degradation occurs, because of the thermal quenching properties of the phosphor, such as luminous decay and color shifting [9]. In order to achieve high efficiency and strong chemical and thermal stability for pc-LEDs, downconversion phosphors have been developed from powders to plates for the Planar-Chip-Level Conversion (PCLC) approach (single crystals (SCs), polycrystalline ceramics, and glasses) [10]. Compared with the above-mentioned converters, single-crystalline converters, with a well-ordered atomic arrangement and dopant distribution, and minimal impurities and defects, possess higher optical transparency, thermal and radiation stability of the optical and luminescent characteristics, and high thermal conductivity (~10 W/mK) [11,12,13,14].

For the development of innovative and energy-efficient solid-state lighting sources, it is urgently needed to design and investigate new high structural quality converters, in the form of single crystals, for the PCLC approach in pc-wLEDs. A variety of Ce^3+^ doped A_3_B_2_C_3_O_12_ (A=Gd, Tb, Y; Lu, Ca B=Al, Ga; C=Ga, Al, Mg, Si) mixed garnets, allow the production of green–yellow–orange-emitting phosphors, which have become a research hotspot [15]. The luminescence properties of the Ce^3+^ activators vary depending on the composition of the garnet matrices and crystallization method, therefore the maxima of the Ce^3+^ emission band can be tuned in the 505–585 nm range [16]. The Lu_3_Al_5_O_12_:Ce (LuAG:Ce) garnet is considered an excellent temperature-stable phosphor for greenish–white lighting emission diodes. Furthermore, the quenching temperature for Ce^3+^ emission in the LuAG host is the highest for all Al-based garnets, with the onset of quenching above 700 K [17]. The greenish emission of the converter reduces the comfort and color fidelity of these white light sources; therefore, to better resemble natural sunlight, the CRI must be increased, and for warmer light applications, the CCT must be decreased. This can be realized by the relative increase in red components in the total emission spectrum, for example by adding an additional thin layer of phosphor converter. The Ce^3+^ emission spectrum in TbAG:Ce is significantly red-shifted compared to the well-known YAG:Ce, due to an increase in crystal field strength in the dodecahedral sites of the garnet lattice, where the Ce^3+^ ions are localized. Such films can be deposited using different techniques such as sputtering deposition [18], electrochemical synthesis [19], sol–gel [20], metal–organic chemical vapor deposition (MOCVD) [21], pulse laser deposition (PLD) [22], and liquid-phase epitaxy (LPE) [23,24] methods. Among the approaches discussed, the LPE technique is a flexible way of producing single-crystalline films (SCFs) for optoelectronic applications, with thicknesses ranging from a few micrometers to 200 μm, with outstanding material quality and reproducibility [25].

One of the first works in this area was from Kundaliya et al. [26], who proposed phosphor-converter production by depositing a YAG:Ce and LuAG:Ce garnet phosphor film epitaxially onto a YAG substrate, to induce yellow and green emission, respectively. Recently, some of our group showed the possibility of the LPE technique for the development of Tb_3_Al_5_O_12_:Ce (TbAG:Ce) single-crystalline film converters for wLEDs [27]. The next step in our research was the development of a composite color converter (CCC), based on the Ce^3+^ doped TbAG:Ce/YAG:Ce film-crystal epitaxial structures [28]. Development of CCCs suggests a few more additional tunable parameters, originating from the single crystal substrate: (i) Ce^3+^ doping concentration in the substrate; (ii) thickness of the substrate. Finally, the combination of the emission coming from the Ce^3+^ doped substrate and film constituents of the color converter under blue or UV LED excitation, allows the production of a wide spectrum of WLEDs similar to the spectrum of natural white light, with enhanced luminous efficacy in comparison with standard photoconverters [27,28].

In this paper, we develop an innovative three-layered composite color converter for white LEDs, based on complex epitaxial structures containing Y_3_Al_5_O_12_:Ce (YAG:Ce) and Tb_3_Al_5_O_12_:Ce (TbAG:Ce) SCFs, steeply grown, using the LPE method, onto an LuAG:Ce SC substrate. The structural luminescence and photoconversion properties of two sets of composite TbAG:Ce SCF/YAG:Ce SCF/LuAG:Ce SC color converters, with variable film thicknesses and Ce^3+^ concentration, in LuAG:Ce substrates (0.01 at. %; 0.2 at. %), are presented in detail as well in this work.

## 2. Materials and Methods

Two sets of TbAG:Ce SCF/YAG:Ce SCF/LuAG:Ce SC (0.01; 0.2 at. %) epitaxial composite structures were prepared using the LPE method at the Chair for Optoelectronic Material of Physics Department of Kazimierz Wielki University in Bydgoszcz, Poland. Namely, the step LPE growth of YAG:Ce and TbAG:Ce SCFs were performed onto (110) oriented LuAG:Ce SC substrates S1 (Ce = 0.01 at. % h = 1 mm) and S2 (Ce = 0.2 at. % h = 1 mm). The substrates were prepared from the respective Czochralski-grown crystals, in the Institute of Scintillation Materials, Kharkiv, Ukraine.

**Figure 1:**
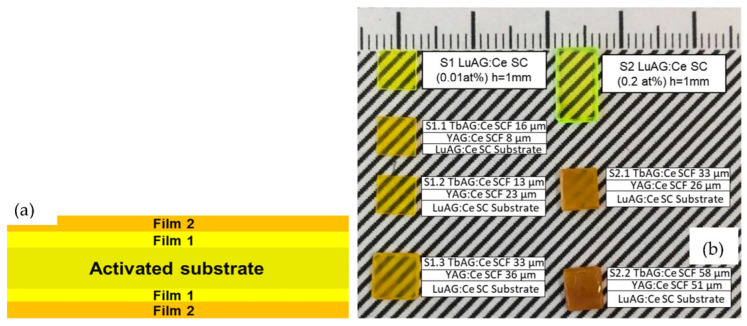
(**a**) Scheme of composite (film–film–substrate) color converter, where film1 is YAG:Ce SCF and film2 is TbAG:Ce SCF. (**b**) Photos of LuAG:Ce substrates (S1 and S2) and LPE-grown TbAG:Ce SCF/YAG:Ce SCF/LuAG:Ce SC epitaxial structures.

It is worth noting that the S1 type of substrates, after commercial mechanical polishing, were additionally chemical–mechanical polished in SiO_2_ slurry; whereas the S2 type of substrates was only mechanically polished, using diamond paste. Their average surface roughness, after the mechanical polishing and additional chemical polishing, was 3.6 nm and 0.3 nm, respectively. Such a difference in the final treatment of the substrates was reflected in the structural quality and optical transparency of the LPE-grown epitaxial structures (Figure 1), due to the partial dissolving of the substrate surface in the melting PbO-B_2_O_3_ flux. For this reason, the optical transparency of the composite converters was notably better in the first set of composite samples (Figure 1).

Materials with low melting points (up to 1000 °C) were used as the flux and provided the necessary level of subcooling of the melt solution. The film’s growth was performed in a Pt crucible from the supercooled melt solution, based on PbO-B_2_O_3_ (12:1 mole/mole) flux and 4N purity garnet-forming oxides: Lu_2_O_3_, Tb_4_O_7_, Gd_2_O_3,_ Al_2_O_3_, and CeO_2_. The content of garnet components in the melt-solution was stoichiometric, calculated regarding the flux composition, see [29] for further detail. Throughout the film growth process, the substrate, horizontally attached to the platinum holder, was rotating at 60–80 rpm in the forming melt-solution, maintaining the growth temperature, T_g_, in the 950–1025 °C range.

The XRD measurements, using a DRON 4 spectrometer (Cu_Kα_ X-ray source) were carried out in the 2θ range, from 91° to 95°, with a step of 0.02°, for the determination of the structural quality and misfit values between the SCF and the YAG:Ce substrate. This low 20-degree XRD technique is typical for the investigation of ion single crystalline film/substrate structures [23,28,29], and allows for the receiving of reflections from the substrate and film in one diffraction pattern.

All optical measurements were performed at room temperature (RT). The absorption spectra were recorded in the 200–1100 nm range, using a Jasco 760 UV-Vis spectrometer. The cathodoluminescence (CL) spectra were measured using an electron microscope SEM JEOL JSM-820, with an added spectrometer, Stellar Net, and TE-cooled CCD detector working in the 200–925 nm range. The PL emission and excitation (PLE) spectra were recorded with an Edinburgh Instruments FS5 spectrofluorometer, equipped with a 150 W xenon lamp as the excitation source. The chromaticity parameters of the samples were determined using AvaSphere-50-IRRAD, in conjunction with a fiber-optic spectrophotometer (AvaSpec-Uls2048-ltec), and a 464 nm blue LED (30 mA, 2.9 V). The color coordinates, correlated color temperature (CCT), and color rendering index (CRI), were calculated using the Avantes software.

## 3. Results and Discussion

### 3.1. Structural Properties

**Figure 2:**
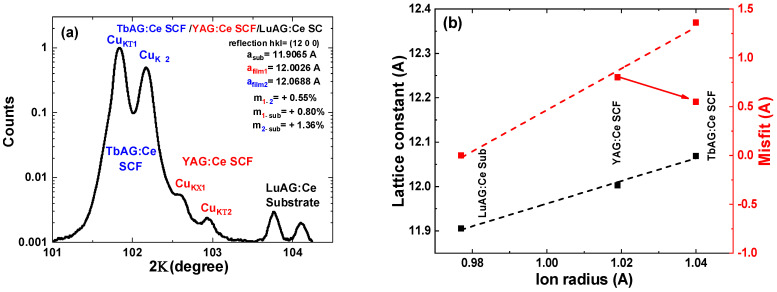
(**a**) XRD pattern of (12 0 0) plane of 23 μm thick TbAG:Ce SCF crystallized on 13 μm thick YAG:Ce SCF grown onto (100) oriented LuAG:Ce substrate. (**b**) Dependence of lattice constant and misfit value on the ionic radius of corresponding cations in dodecahedral positions of garnet lattice (dashed line, in comparison with LuAG:Ce; arrow, in comparison with YAG:Ce SCF). The accuracy of determining the lattice constant is ±0.0002 Ǻ.

The X-ray phase analysis confirmed that all the synthesized compounds were single-phase samples, containing only the garnet phases, and no XRD reflections corresponding to any other phase were detected. In particular, the XRD pattern of the TbAG:Ce SCF/YAG:Ce SCF/LuAG:Ce SC CCC is shown in Figure 2a. The lattice mismatch between the substrate and the film was estimated using the formula m = (a_film_ − a_sub_)/a_sub_ × 100%. The measured lattice constant of the LuAG substrate was a_s_ = 11.9065 Å, when the lattice constants of the YAG:Ce and TbAG:Ce films were determined as a_film1_ = 11.9082 Å and a_film2_ = 11.0609 Å, respectively. The dependence of the lattice constant and misfit values on the ionic radius of cations in dodecahedral positions, is shown in Figure 2b. It can be seen that there is an increase in the unit cell parameter with the ionic radius of the {Lu^3+^, Y^3+,^ Tb^3+^} cations, residing in dodecahedral sites on the garnet unit cell, which determines the peak positions of the XRD reflections for these garnets. It is noticeable that the dependencies are linear, and consistent with Vegard’s law [30].

The obtained structural data for {R}_3_Al_5_O_12_:Ce; R = Lu, Y, Tb, constituent parts are systematized in Table 1. It should be noted here that a TbAG:Ce film cannot be directly grown on an LuAG:Ce substrate, due to a misfit exceeding 1%, which is the limiting value for successful crystallization of single-crystal films by the LPE technique [31]. Therefore, the YAG:Ce film occurs here as a specific buffer layer for the following LPE growth of TbAG:Ce SCFs.

### 3.2. Absorption and Photoluminescent Properties of Epitaxial Structures

**Figure 3:**
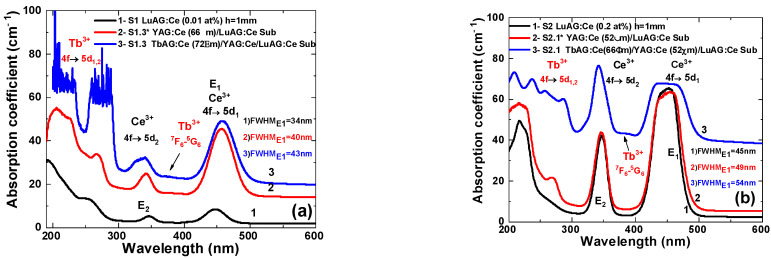
RT absorption spectra of the TbAG:Ce SCF/YAG:Ce SCF epitaxial structures (S1 and S2) grown onto LuAG:Ce substrates, with different Ce^3+^ concentration of 0.01 at. % (**a**), and 0.2 at. % (**b**).

Figure 3a,b demonstrates the absorption spectra of the TbAG:Ce SCF/YAG:Ce SCF/LuAG:Ce SC epitaxial structures of the S1 and S2 series, respectively, with comparable film thicknesses. The absorption spectra were measured after the growth of the next layer of CCCs. The spectra for the LuAG:Ce substrates (Figure 3a,b, curve 1) show two broad absorption bands, located at ~445 nm and ~340 nm, which are ascribed to the allowed 4f→5d_1_ (E_1_) and 4f→5d_2_ (E_2_) transitions of the Ce^3+^, respectively, in the garnet phase. The absorption coefficients for the Ce^3+^ absorption transitions significantly vary between the two substrates, due to the different Ce content in these samples. Furthermore, a wide absorption band, centered at 254 nm, is observed. This band belongs to the ^1^S_0_-^3^P_1_ transition of Pb^2+^ ions, coming into the SCF from the PbO based flux during LPE growth.

The YAG:Ce SCF/LuAG:Ce SC epitaxial structure (Figure 3a,b, curve 2) shows up the superposition of Ce^3+^ absorption bands in the YAG:Ce film and LuAG:Ce crystal. The substitution of Lu for Y in aluminum garnets changes the crystal field strength, and consequently shifts the 4f→5d_1_ (E1) and 4f→5d_2_ (E2) absorption bands to lower and higher energies, respectively [33,34].

The imposition of Ce^3+^ absorption bands from both garnet matrices results in a significant broadening of the main E_1_ absorption band (indicated in Figure 3), which is advantageous for use with commercially available blue LEDs, for wLEDs development. In the case of three layered TbAG:Ce SCF/YAG:Ce SCF/LuAG:Ce SC epitaxial structures, besides overlapping Ce absorption bands, shifted relative to themselves, the absorption spectra contain sets of bands belonging to the transitions of Tb^3+^ ions. Namely, the bands centered around ~240 nm and ~290 nm, belong to the low spin-allowed (LS) 4f→5d_2_ and 4f→5d_1_ absorption transitions of Tb^3+^ ions, respectively. The peak at 373 nm is ascribed to the ^7^F_6_→^5^G_6_ absorption transition of Tb^3+^ cations. It should be noted that for curve 3 in Figure 3b, the background rises, due to the weak transparency of the sample, which may be due to the light scattering in the case of partially dissolving the SCFs surface in their preparation. The amplitudes of the 4f→5d_1,2_ Tb^3+^ absorption bands, centered at ~275 nm and ~225 nm, are saturated, due to reaching the limit of the spectrometer and the low transparency of the samples in that region.

**Figure 4:**
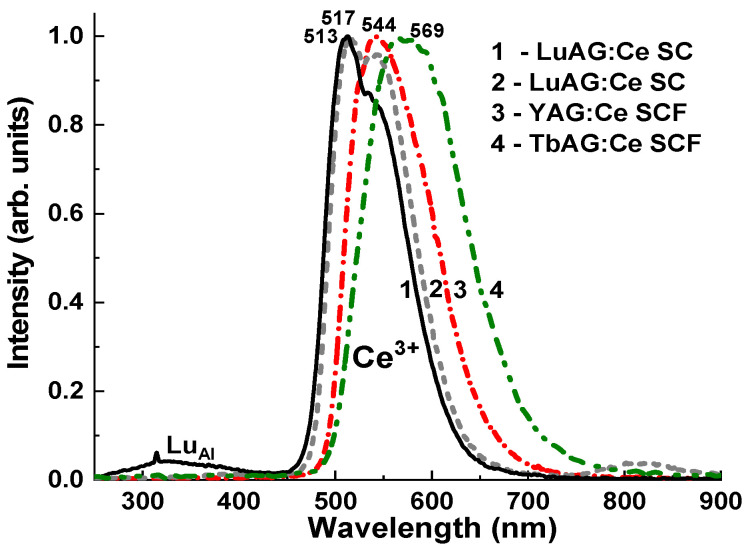
CL spectra of the LuAG:Ce (0.01 at. %) SC (1) and LuAG:Ce (0.2 at. %) SC (2) substrates, and the YAG:Ce SCF/LuAG:Ce SC (S1.3*) (3), and the TbAG:Ce SCF/YAG:Ce SCF/LuAG:Ce SC (S1.3) (4) epitaxial structures.

The normalized CL spectra of the CL spectra of LuAG:Ce (0.01 and 0.2 at. %) SC substrates and YAG:Ce SCF/LuAG:Ce SC and TbAG:Ce SCF/YAG:Ce SCF/LuAG:Ce SC epitaxial structures are shown in Figure 4. The observed luminescence bands in the visible range peaked at 513 nm and 517 nm in the substrates and 544 nm and 569 nm for the LuAG:Ce and TbAG:Ce SCFs, as part of the composite converters is related to the 5d^1^→4f (^2^F_5/2_; ^2^F_7/2_) transition of Ce^3+^ ions in the garnet hosts. The observed shift in the position of the Ce^3+^ emission bands in the mentioned SCFs, is due to the change in the crystal field strength in the crystal lattice of the LuAG, YAG, and TbAG garnet hosts. The intensity crystal field strength in the dodecahedral sites of the garnet lattice directly depends on the R^3+^-O^2−^cation–anion distances. As the ionic radius of R^3+^ increases (e.g., Lu^3+^ is substituted with Y^3+^ and Tb^3+^), the corresponding cation–anion distances become smaller, and the crystal field strength is larger. Namely, changing the ion radius from 0.977 Ǻ for Lu^3+^, to 1.019 Ǻ for Y^3+^, and to 1.04 Ǻ for Tb^3+^, in the dodecahedral position (CN = 8) of the garnet host (Table 1), leads to a notable increase in the crystal field strength. The higher crystal field strength causes the lowering of the excited state, 5d^1^, of Ce^3+^ ions, and the corresponding shift of the maximum Ce^3+^ emission band shifts to the red range.

In the case of the LuAG:Ce (0.01 at. %) and LuAG:Ce (0.2 at. %) SC substrates, the red-shift of the CL spectra from 513 nm to 517 nm, is due to the significantly large content of Ce^3+^ ions in the last crystals and respective increase in the crystal field strength caused by the substitution of the Lu^3+^ cation by the significantly larger Ce^3+^ ions (1.143 Ǻ). The emission band of LuAG:Ce (0.01 at. %) in the UV range is caused by the luminescence of Lu_Al_ antisite defect related centers (see [35,36] for details).

**Figure 5:**
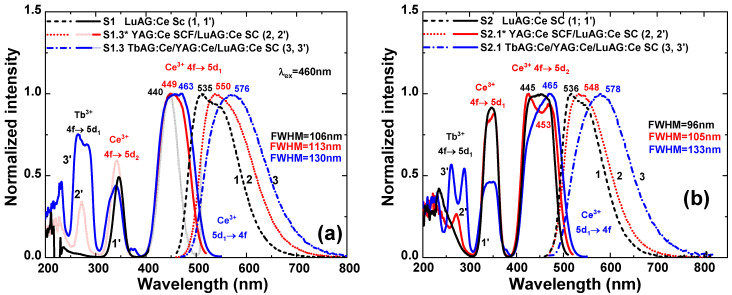
RT PLE spectrum of the Ce^3+^ luminescence at 560 nm, and PL spectrum excited at 460 nm for the S1.3 (**a**) and S2.1 (**b**) composite converters.

The RT photoluminescence emission (PE) and excitation (PLE) spectra of the TbAG:Ce SCF/YAG:Ce SCF/LuAG:Ce SC composite color converter, are compared in Figure 5 with its YAG:Ce SCF/LuAG:Ce SC and LuAG:Ce constituent parts. The PLE spectra of the substrates and the composite color converter (dashed curves), for the Ce^3+^ emission peak maxima for each layer, are mostly identical with their absorption spectra belonging to Ce^3+^ and Tb^3+^ ions absorption transitions (Figure 3). The presence of Tb^3+^ related bands in the excitation spectra of Ce^3+^ emission is associated with an efficient energy transfer (ET) from the main Ce^3+^ excitation band, related to the 4f-5d_1_ transition, in the composite converter is redshifted about 21–23 nm, and centered at 463 nm and 465 nm for the S1.3 and S2.1 samples (Figure 5a,b, curves 3, 3′). As a result, the position of the excitation maximum overlaps perfectly with 464 nm commercial blue LEDs.

The PL emission spectra of the layer-by-layer epitaxial structures under study, excited at 460 nm, are shown in Figure 5 (solid curves). In comparison to the typical emission of LuAG:Ce (0.01 and 0.2 at.%) SC substrates, with maxima around 535 and 536 nm, related to the 5d_1_(E_2g_)→ 4f(^2^F_5/2_) and 5d_1_(E_2g_)→4f(^2^F_7/2_) transitions of Ce^3+^ ions (Figure 5a,b, respectively (curve 1)), the emission spectra of the YAG:Ce SCF/LuAG:Ce SC composite converters (Figure 5a,b, curve 2) represent the overlapped broad yellow–orange emission of Ce^3+^ ions, both in the LuAG:Ce substrates and the YAG:Ce film. This allows for a broadening and red-shifting of the emission band, with a peak around 550 nm, and increasing of the FWHM from 96–106 nm to 105–113 nm, for the S1 and S2 group samples, respectively, in comparison with the LuAG:Ce substrates. Finally, for the three-layered TbAG:Ce SCF/YAG:Ce SCF/LuAG:Ce SC composite color converters, the emission maxima are located around 576–578 nm, with an increased FWHM even up to 133 nm (Figure 5a,b, curve 3). The emission red-shift is related to increased crystal field splitting and consequently the larger split of 5d energy level of Ce^3+^ ions and the increased stokes shifts due to the increase in {Lu→Y→Tb}ions radius in dodecahedral sites of the garnet structure (Table 1) [31,32]. Furthermore, the concentration of Ce^3+^ in the garnet host affects the position of the half-width and intensity of the Ce^3+^ emission bands in films and substrate, allowing valuable control of the photo-conversion properties of pc-WLEDs.

### 3.3. Photoconversion Properties

**Figure 6:**
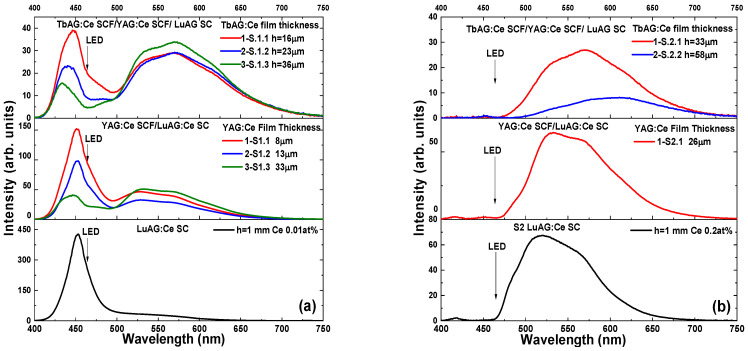
Emission spectra for the light output of composite color converters with different film thicknesses. (**a**) S1 group, (**b**) S2 group, under 464 nm blue LED excitation (mentioned with the arrows).

In order to screen the optimal spectral composition, the YAG:Ce and TbAG film phosphors were combined with LuAG:Ce SC with different Ce^3+^ concentrations, to investigate their photoconversion properties under the excitation of the blue LED chip OSRAM LBE 6SG, driven with a forward-bias voltage of 2.9 V and current of 30 mA. Figure 6 displays the emission spectra for the light output of the wLED prototypes based on the three-layered CCCs. The spectra were measured firstly for the S1 and S2 LuAG:Ce substrates, and then for each subsequently LPE-grown SCF with different thicknesses, first with YAG:Ce and then with TbAG:Ce on it. The band around 464 nm is attributed to the emission of the blue LED transmitted through the sample, and the broad yellow emission band of Ce^3+^ ions emanating from each layer of the composite converters. It is important to note here, that for the LuAG:Ce SC substrates (Figure 5, the bottom section), at rising Ce concentration, the intensity of the blue component decreases, while the yellow component increases. Furthermore, in the case of the S2 substrate, the blue component is almost absent. According to the absorption spectra in Figure 3, this can be explained by a higher blue light absorption by the S2 substrates in comparison with S1.

The imposition of a thin film on a substrate clearly alters the spectrum of a composite converter. If one or two films are added, there is a simultaneous change in the intensity and position of the blue band, as well as a broadening and red-shifting of the yellow emission components emanating from the basic substrate and the film. This approach in engineering the emission spectrum of a white LED, allows for the correction of the green gap in the spectrum, thereby bringing it closer to the spectrum of a natural source, in a peculiar way. It is worth noting that the color coordinates of different structures can be closest to the blackbody line, and can be used for neutral white light illumination by changing the thicknesses or Ce concentration in each component of the three-layer composite converter.

**Figure 7:**
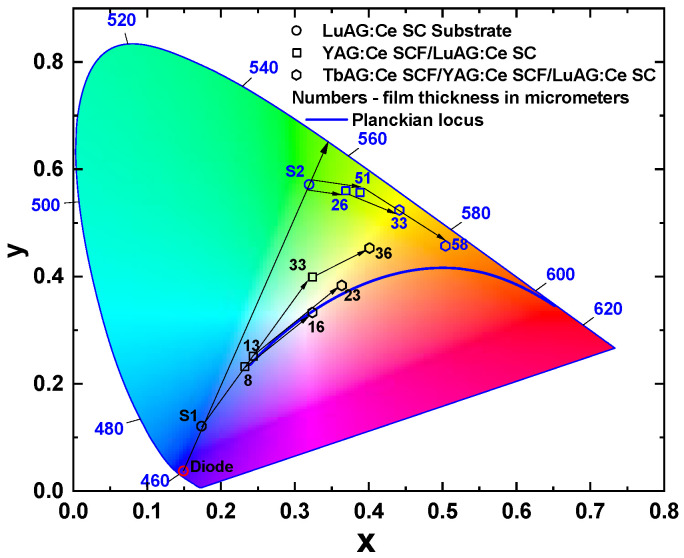
Color coordinates of wLED prototypes, with a composite color converter, on CIE-1931 chromaticity diagram.

The Commission Internationale De L’Eclairage (CIE) chromaticity coordinates of wLEDs fabricated using the LuAG:Ce SC substrate and YAG:Ce and TbAG:Ce SCFS, are shown in Figure 7. The circles in the diagram show the color coordinates that correspond to LuAG:Ce substrates with different cerium concentrations. As can be seen, the coordinates of the samples lie on the trend line passing through the blue LED point and the green area of the diagram. Substrates with such a concentration were specially chosen, so that their color coordinates were in diametrically opposite areas of the diagram, to demonstrate a possible change in trends when applying subsequent films.

The angle trend line (formed and indicated on the diagram by a square symbol) towards the yellow area can be seen after the crystallization of the YAG:Ce SCFs onto the LuAG:Ce SC substrates (S1 and S2 samples). However, the color coordinates of these points are far from the Planckian locus of the blackbody (blue solid line in Figure 7). The color coordinates of the final structures, TbAG:Ce SCF/YAG:Ce SCF/LuAG:Ce SC, for both groups crystallized in this work are shown in the diagram by the hexagon symbol. It is worth noting that the color coordinates of different structures can be closest to the blackbody line, and can be used for neutral white light illumination by changing the thicknesses of the film phosphors. Such an approach enables the reduction in the radiation’s correlated color temperature (CCT), and the optimization of its color rendering index (CRI), as shown in Table 2 and Table 3. Namely, the color coordinates of the S1.1 sample of CCC were dramatically shifted from S1.1* (0.232, 0.232), which is inappropriate for wLEDS, to near theoretical white coordinates (0.325, 0.333), with the highest obtained CRI. The photoconversion parameters of the wLED prototypes based on TbAG:Ce SCF/YAG:Ce SCF/LuAG:Ce SC CCCs, with various film thicknesses and LuAG:Ce doping concentration, are listed in Table 2 and Table 3.

## 4. Conclusions

In summary, we have developed a new type of TbAG:Ce film/LuAG:Ce film/LuAG:Ce crystal composite color converter, using the LPE growth method. When compared to the greenish light from the LuAG:Ce crystal substrate, the Ce^3+^ emission in the composite epitaxial structures is significantly broadened and red-shifted, yielding additional yellow/orange light. Therefore, the color consistency of white LEDs may be significantly enhanced by employing single crystalline films where the phosphor materials are uniformly deposited onto a single crystalline substrate. This technology also enables also the control of the color temperature, and minimizes wLED production variance.

We have found that the combination of different LuAG:Ce substrates with YAG:Ce and TbAG:Ce single crystalline films, enable precision tuning of the white light tones, from cold white/daylight white (CCT > 6000 K) to neutral white (6000 K > CCT > 3300 K). The ideal white color was achieved for S1.1 TbAG:Ce film/LuAG:Ce film/LuAG:Ce crystal with TbAG:Ce and YAG:Ce SCF with thicknesses of 16 µm and 8 µm, respectively, under 464 nm LED excitation.

## Figures and Tables

**Table 1:** The values of lattice constant and misfit of garnet SCFs (misfit values were calculated for each next layer of the composite) with the corresponding ionic radius of aluminum garnet forming ion in dodecahedral position (Lu, Y, Tb) [32].

Composition	Ionic Radius CN = 8, Ǻ	Lattice Constant, Ǻ	Misfit, %
LuAG:Ce SC substrate	0.977	11.9065	-
YAG:Ce SCF	1.019	12.0026	0.8
TbAG:Ce SCF	1.04	12.0688	0.55

**Table 2:** Photoconversion properties of S1 group composite color converters.

Sample	Film h, µm	x	y	CCT, K	CRI
LuAG:Ce SC substrate					
S1.	0	0.170	0.104	N/A	N/A
YAG:Ce SCF/LuAG:Ce SC □					
S1.1*	8	0.232	0.232	N/A	N/A
S1.2*	13	0.244	0.251	N/A	N/A
S1.3*	33	0.322	0.394	5839	65
TbAG:Ce SCF/YAG:Ce SCF/LuAG:Ce SC ⬢					
S1.1	16	0.325	0.333	5852	77
S1.2	23	0.369	0.388	4375	71
S1.3	36	0.397	0.446	4032	66

**Table 3:** Photoconversion properties of S2 group composite color converters.

Sample	Film h, µm	x	y	CCT, K	CRI
LuAG:Ce SC substrate					
S2	0	0.319	0.571	N/A	27
YAG:Ce SCF/LuAG:Ce SC □					
S2.1*	26	N/A	N/A	N/A	N/A
S2.2*	51	0.391	0.556	N/A	39
TbAG:Ce SCF/YAG:Ce SCF/LuAG:Ce SC ⬢					
S2.1	33	0.442	0.523	3672	51
S2.2	58	0.504	0.456	2480	71

## Data Availability

The data presented in this study are available on request from the corresponding author. The data are not publicly available due to patent restrictions.
